# Clinical characteristics, treatment, and long-term outcomes in children suffering from benign convulsions with mild gastroenteritis: a retrospective study

**DOI:** 10.1186/s12887-020-02406-0

**Published:** 2020-11-10

**Authors:** Xiao-sheng Hao, Jian-min Liang, Xue-mei Wu, Yun-peng Hao, Jiang-tao Wang, Song-yan Liu

**Affiliations:** 1 The First Hospital of Jilin University, 130021 Changchun, Jilin China; 2 The China-Japan Union Hospital, 130033 Changchun, Jilin China

**Keywords:** Benign convulsions with mild gastroenteritis, rotavirus, lumbar puncture

## Abstract

**Background:**

Benign convulsions with mild gastroenteritis (BCWG) is a common condition in children in Asia and is generally not associated with pH or electrolyte imbalances. When BCWG is diagnosed, a lumbar puncture is usually recommended to rule out potential intracranial infections. This study examined the clinical characteristics of BCWG and evaluated the necessity of lumbar puncture.

**Methods:**

Medical records of children admitted to the First Hospital of Jilin University with BCWG between January 2018 and May 2019 were reviewed and analyzed. Children were stratified by rotavirus positivity or lumbar puncture status. Clinical characteristics and long-term outcomes were compared between groups.

**Results:**

A total of 51 children were included in the analyses (55.1% rotavirus [HRV] positive). The average age of convulsion onset was 21.12 ± 7.44 months, the male-to-female ratio was 1.8:1, and convulsions occurred primarily between October 2018 and April 2019. The main clinical presentations of BCWG were convulsions, vomiting, diarrhea, and fever. Convulsions occurred predominantly two days after diagnosis of gastroenteritis, were mainly generalized tonic-clonic with 88.2% of children having ≤ 3 convulsions per episode, and had a mean duration of 2.0 minutes (interquartile range [IQR]: 1.0, 3.0). Children with BCWG had mild metabolic acidosis (HCO_3_^−^ 17.82 ± 3.63 mmol/L) with an elevated anion gap (AG; 20.98 ± 3.00 mmol/L), mild hyponatremia (134.56 ± 2.85 mmol/L), and slightly increased levels of creatine kinase myocardial band (CKMB). HRV + children had more severe acidosis and higher CKMB levels. Cerebrospinal fluid (CSF) samples collected via lumbar puncture were normal. No developmental abnormalities were noted as assessed by the Social Life Ability Scale.

**Conclusions:**

BCWG is a situation-related seizure, with clinical presentations of tonic-clonic or focal convulsions and mild gastroenteritis (vomiting, diarrhea). Mild metabolic acidosis and hyponatremia may exist. The prognosis of the disease is favorable; lumbar puncture and long-term antiepileptics are unnecessary and should not be recommended.

## Background

Benign convulsions with mild gastroenteritis (BCWG or CwG) is a type of benign, infantile convulsion [1]. BCWG was first reported by Morooka (1982), and presentation of the condition includes mild gastroenteritis with afebrile convulsions (which can be generalized tonic-clonic seizures or partial seizures, mild dehydration with no electrolyte imbalance, acidosis, or hypoglycemia), with normal interictal electroencephalogram (EEG), normal cerebrospinal fluid (CSF) and normal brain imaging [2, 3]. BCWG is most commonly reported in children aged six months to three years and in East Asian countries, suggesting that a risk factor for BCWG may be ethnic. The exact incidence rate is difficult to estimate. However, the incidence rate of BCWG associated with rotavirus enteritis is estimated to be 2–3%, and the incidence of BCWG in North China is 1.78% [4, 5].

Although BCWG has been recognized as benign partial epilepsy in infancy, the International League Against Epilepsy (ILAE) has not officially classified BCWG [6]. At present, BCWG is generally considered to be a benign situation-related seizure rather than epilepsy [7]. The disease is often described as an afebrile seizure associated with mild gastroenteritis, and no abnormalities in laboratory test results have been reported. Lumbar punctures, to eliminate infectious brain diseases, are often recommended [2]. However, in our clinical practice, we have observed mild acidosis and electrolyte imbalance in children with BCWG, whereas the CSF sampled via lumbar puncture showed no abnormalities. In this retrospective study, we aim to examine the clinical presentation of BCWG and the necessity of lumbar punctures.

## Methods

### Patients and data collection

In this retrospective study, we reviewed the medical records of children who were hospitalized for BCWG in the Department of Pediatric Neurology, First Hospital of Jilin University between January 2018 and May 2019. This analysis did not include cases of convulsions caused by psychomotor developmental disorders, epilepsy, encephalitis, meningitis, or encephalopathy.

Medical chart information reviewed in this study included medical history (clinical presentation [based on parents’ reports], past treatments, and outcome), laboratory tests including routine blood test, routine stool analysis, blood gas analysis, serum electrolytes, blood glucose, myocardial enzymes tests (creatine kinase [CK] and creatine kinase myocardial band [CKMB]), human rotavirus antigen (HRV), and C-reactive protein (CRP). Lumbar punctures, computed tomography (CT) or magnetic resonance imaging (MRI), and long-term electroencephalogram (EEG) results were analyzed when available.

Children included in this analysis were stratified by their HRV status or lumbar puncture completion status. The children were followed up by telephone or outpatient visits for 7–24 months after discharge from the hospital. The Social Life Ability Scale, a questionnaire based on the Japanese S-M social life ability test and modified for Chinese children between the ages of six months and 15 years, was used to assess the developmental and functional status of children included in this study [8]. Depending on age, the children, parents, or caretakers rated their functional status in the following domains: self-help, locomotion, occupation, communication, socialization, and self-direction.

All the children in the study were under the age of 16. Written informed consent was obtained from a parent or guardian on behalf of all individual participants. This study was approved by the ethics committee of the First Hospital of Jilin University.

### Statistical Analysis

Continuous data with normal distributions were presented as mean ± standard deviation (SD) and continuous data with abnormal distributions were presented as median values (interquartile range [IQR]). Significance between groups was ascertained using unpaired t-tests for data with normal distributions, the Wilcoxon rank sum test was used for data with non-normal distributions, and the Chi-squared test was used for categorical data. An α value of < 0.05 was considered to denote statistical significance. Statistical software (SPSS, version 21.0) was used for all analyses.

## Results

### Patients

The present retrospective study included 51 children with BCWG who were hospitalized between January 2018 and May 2019. Demographic information of the patients and disease characteristics stratified by HRV status are summarized in Table 1. Briefly, the 51 children with BCWG included in the study were 33 boys and 18 girls (male to female ratio of 1.8:1). The age of onset ranged between five and 36 months (mean 21.1 ± 7.4 months), including two children under 12 months, 22 between 13 and 18 months, 12 between 19 and 24 months, 10 between 25 and 30 months, and five between 31 and 36 months. It is noted that although the disease can occur throughout the entire year, most children (90.2%, 46/51) experienced convulsions between October and April (15 children experienced convulsions between March and May, two between June and August, 10 between September and November, and 24 between December and February). The mean time between seizure onset and the first hospital visit was 2.0 ± 0.8 days (range, 1.0–3.0 days). Before admission to the hospital, none of the children included in this study had a history of convulsions, and no abnormalities at birth or during development were noted. Except for one child with a family history of febrile convulsion and another child with a family history of epilepsy, all children included in this study had no previous medical history or family history of convulsion.

**Table 1:** Patient demographic information and characteristics stratified by HRV status

	Total (*N* = 51)	HRV positive (*N* = 27)	HRV negative (*N* = 22)	*P*-value
Age of onset (months)	21.12 ± 7.44	21.37 ± 8.19	21.00 ± 6.93	0.824
Female, n (%)	33 (64.7%)	18 (66.7%)	14 (63.6%)	0.825
Clinical presentation				
Vomiting, (times/day)	4 ± 2	4 ± 2	4 ± 3	0.953
Diarrhea (times/day)	3.0 (2.0, 4.0)	3.0 (1.8, 4.0)	3.0 (2.0, 4.8)	0.937
Duration of convulsion (min)	2.0 (1.0, 3.0)	2.0 (1, 3.5)	2.0 (1.0, 3.0)	0.853
Interval between diagnosis of BCWG and convulsion (days)	2.0 (1.0, 2.0)	2.0 (1.0, 2.0)	1.0 (0.5, 2.5)	0.307
Duration of hospital stay (days)	7.2 ± 3.1	8.0 ± 3.2	6.5 ± 2.6	0.093
Routine blood test laboratory results				
pH	7.34 ± 0.06	7.33 ± 0.06	7.35 ± 0.07	0.311
PCO_2_ (mmHg)	33.37 ± 5.94	31.55 ± 6.02	34.78 ± 5.30	0.102
HCO_3_^−^ (mmol/L)	17.82 ± 3.63	16.67 ± 2.97	18.83 ± 3.99	0.024*
BE (mmol/L)	-6.87 ± 4.13	-7.85 ± 3.64	-6.08 ± 4.56	0.150
Na^+^(mmol/L)	135.0 (133.8,136.0)	135.0 (133.2, 136.0)	134.8 (133.88, 135.93)	0.695
K^+^ (mmol/L)	4.08 ± 0.45	4.09 ± 0.50	4.09 ± 0.41	0.943
Cl^−^ (mmol/L)	102.0 (99.0, 103.0)	102.0 (102.0, 103.0)	100.2 (98.8, 102.0)	0.033*
Ca^2+^ (mmol/L)	1.25 ± 0.68	1.27 ± 0.06	1.23 ± 0.07	0.037*
Glucose (mmol/L)	4.10 (3.70,4.63)	4.10 (3.70, 4.60)	4.10 (3.75, 4.75)	0.479
Lac (mmol/L)	1.60 ± 0.82	1.61 ± 0.81	1.59 ± 0.84	0.789
CK (U/L)	99.0 (70.5, 126.5)	94.0 (69.5, 142.0)	104.0 (69.5, 126.5)	0.910
CKMB (U/L)	51.0 (35.7, 80.3)	60.9 (45.2, 91.4)	46.0 (31.8, 64.3)	0.036*
WBC (× 10^9^/L)	7.5 ± 3.0	7.9 ± 2.9	7.2 ± 3.1	0.294
NE (%)	0.51 ± 0.19	0.56 ± 0.20	0.45 ± 0.17	0.051
LY (%)	0.41 ± 0.18	0.36 ± 0.18	0.45 ± 0.17	0.057
AG (mmol/L)	20.94 (18.19, 23.68)	20.94 (19.24, 23.19)	21.30 (17.79, 25.64)	0.769
CRP (mg/dL)	2.8 9 ± 3.51	2.96 ± 1.93	2.67 ± 2.06	0.304

*AG* anion gap; *BE* bases excess; *CK* creatine kinase; *CKMB* creatine kinase myocardial band; *CRP* C-reactive protein; *HRV* human rotavirus antigen; *Lac* lactate; *LY* lymphocyte; *NE* neutrophil elastase; *WBC* white blood cell

* Significant difference (*p* < 0.05) was noted between the groups

When stratified by HRV status (two children without HRV status information were excluded from subsequent comparisons), no significant difference in patient characteristics was noted between the HRV + group (*n* = 27) and the HRV- group (*n* = 22).

### Clinical presentation of mild gastroenteritis

In this study, the frequency of diarrhea and vomiting, both symptoms of mild gastroenteritis, were analyzed. Most children (94.1%, 48/51) experienced mild to moderate diarrhea, defined as having fewer than 10 loose or watery stools per day. The mean frequency of diarrhea was 3.0 (2.0, 4.0) times per day. Vomiting was noted in 45 (88.2%) children, with a mean frequency of vomiting 3.7 ± 0.3 (range, 0–10) times per day; the majority of children (42/45) vomited less than five times per day. Moderate or severe dehydration was not observed in any children. No significant difference in clinical presentation of mild gastroenteritis was noted between the HRV + and HRV- groups.

### Clinical presentation of convulsions

Types of convulsion were determined based on descriptions provided by the parents or observations by treating physicians. Among the 51 children, 17 (36.4%) had tonic seizures, 30 (63.6%) had tonic-clonic seizures, and no focal seizures were noted.

The median frequency of convulsion in a single episode was 2.1 times (range, 1‒5). Most children (45/51) experienced three or fewer convulsions during an episode, with 13 children experiencing one, 25 experiencing two, seven experiencing three, three experiencing four, and three experiencing five convulsions.

The mean duration of convulsions was two minutes (range 0.5–10). In most children (80.4%, 41/51), convulsions lasted for less than three minutes.

No significant difference in the clinical presentation of convulsion was observed between the HRV + and HRV- groups.

### Interval between diagnosis of gastroenteritis and onset of convulsions

In this study, the mean time interval between clinical presentation of gastroenteritis and the onset of convulsion was two days (range 1–5). In most cases, convulsions occurred after gastroenteritis symptoms had presented (49/51), usually within two days (78.4%, 40/51); however, two children experienced a convulsion before showing symptoms of gastroenteritis.

### Other clinical presentations

Fever did not occur in 29 children included in this study, while the other 22 children experienced fever, with a mean peak body temperature of 38.6 ± 0.1ºC (range, 37.5–39.7ºC ) within the first three days of gastroenteritis presentation. Convulsions did not occur simultaneously with fever.

All children were in a good mental state after the convulsions were resolved, with no subsequent dyskinesia and/or language disorder. No apparent abnormalities were found in physical examinations of the nervous systems.

### Laboratory tests

Laboratory test results are stratified by HRV status and summarized in Table 1. All children had normal liver and kidney function. While white blood cell (WBC) counts were normal, an increase in neutrophil count was noted. The blood HCO_3_^−^ level was significantly lower while Cl^−^ and Ca^2+^ levels were significantly higher in the HRV + group than in the HRV- group. No occult blood or WBCs were found in the stool samples, and the stool culture results were negative. The CKMB level was significantly higher in HRV + children than in HRV- children.

### CSF examination, imaging analysis, and long-term EEG results

In this study, 24 children (11 boys and 13 girls) underwent lumbar punctures. CSF appeared to be clear with no abnormalities observed, and the CSF pressure was normal in all cases.

Either cranial CT or MRI scans were performed on all children included in the analysis with no abnormal findings. Thirty-nine children were monitored with interictal EEG tests for two to six days after the onset of convulsion. Small sharp spikes of sleep (SSS) in the frontal, temporal, and central parietal regions were detected in nine children. No abnormalities in the EEG tests were observed at the three-month follow-up examination. All other children showed normal EEG results.

### Treatment and follow-up

All the children were treated for symptoms including deficiency in electrolytes. Additionally, phenobarbital sodium (5 mg/kg, intramuscular administration) was used to treat 28 children within 24 hours of admission, and subsequently every 8–12 hours with the same dosage. Phenobarbital was administered two or three times to each child and was discontinued if no convulsion occurred on the second day of admission. Out of the 51 children included in the study, two experienced a seizure episode after hospitalization and were treated with coloclysis (10% chloral hydrate, 0.5 mL/kg). None of the patients were prescribed antiepileptics after being discharged.

The mean follow-up period was 12 months (range, 7–24). Out of the 44 children who completed the follow-up period, two had additional tonic-clonic seizures occurring 12 and 13 months after the discharge date and were diagnosed as BCWG cases. No difference in daily function, as assessed with the Social Life Ability Scale, was observed between children who underwent lumbar punctures (*n* = 24) compared with those who did not (*n* = 27).

## Discussion

BCWG is a condition with the highest incidence rate in children between one and two years of age [9]. Consistent with previous reports, we found that the age of onset is approximately 21 months, and that BCWG occurs more commonly in boys than girls [4, 10]. We also observed that BCWG occurs more often in autumn, winter, and spring months, which may be related to the high incidence of gastroenteritis in these seasons.

Previous studies have shown a favorable prognosis for BCWG [7, 10]. The recurrence rate of BCWG has been reported to range from 7.7–16.8% in earlier studies [7, 11]. Here, we report similar favorable long-term outcomes: two out of 44 children who completed long-term follow-ups had recurrent seizures that did not develop into epilepsy. All children showed normal psychomotor development. Therefore, prescription of long-term antiepileptics is not recommended [12].

Although BCWG has not been shown to cause an electrolyte imbalance, we observed symptoms indicative of mild metabolic acidosis; namely, a decrease in blood pH, HCO_3_^−^, and bases excess (BE) levels [2]. The increase in anion gap (AG) with consistent blood chlorine levels likely resulted from an increase in acidic metabolites. The decrease in PCO_2_ may have resulted from respiratory alkalosis. It is possible that oxygen deprivation may occur due to the repetitive convulsions and subsequent hyperventilation after the convulsions have subsided. As with previous studies, we also observed mild hyponatremia in children with BCWG [9, 11, 13, 14]. Overall, we found a mild imbalance of blood electrolytes in children with BCWG.

BCWG seizures are often clustered, with generalized tonic-clonic seizures forming the most common type of seizure cluster, although focal or secondary generalized seizures are also observed [7, 12, 15]. Consistent with previous reports, we found that tonic or generalized tonic-clonic seizures were the most common types of seizures associated with BCWG; however, it is very likely that BCWG originated as a focal event as spikes were observed in the EEG tests that were localized to various regions of the cortex. Other seizure characteristics, including convulsion frequency and duration, were similar to previous reports [11, 16]. We found that treatment of BCWG with sedatives, preferably phenobarbital (over diazepam) was effective [17, 18]. Convulsions resolved and treatment with sedatives was usually discontinued within 24 hours of admission to the hospital. Transient side effects, including mild lethargy and gait abnormalities, were not common.

Consistent with previous observations, we found that convulsions often occurred two days after the diagnosis of gastroenteritis [19]. However, we also observed the occurrence of convulsions before or without clinical presentation of gastroenteritis [20].

Although approximately 43% of children included in the study had fever, we did not find a correlation between fever and convulsions, which is consistent with the finding by Zifman et al. (2011) that fever did not affect seizure characteristics [14]. The mechanism by which mild gastroenteritis induces convulsions remains to be identified.

Inflammatory responses induced by gastroenteritis and potential cardiac injuries were indicated by the elevated levels of neutrophils and CKMB, respectively, which may result from enterovirus infections. Norovirus and rotavirus are often detected in stool samples collected from children with BCWG. Rotavirus infections have become less common since vaccination against the virus has been incorporated into the recommended vaccination schedule. As a result, gastroenteritis caused by norovirus infection and norovirus-related BCWG has become more common [19]. In contrast, possibly due to the low rotavirus vaccination rate in the region, we found that 55.1% of children included in this study were HRV+. Wang and Zhou (2013) have reported a similar rotavirus infection rate (53.9%) in a retrospective study of 48 children with BCWG, indicating rotavirus infections remain a key risk factor for BCWG [15]. Additionally, metabolic acidosis was more pronounced in HRV + children, and damage to the myocardium as indicated by increased CKMB levels may be more pronounced in these children. These observations underscore the importance of vaccination against rotavirus in infants and children.

We do not recommend lumbar puncture, an invasive diagnostic test, for the diagnosis of BCWG, as it is unlikely that it will provide additional evidence for the diagnosis of BCWG. Lumbar puncture is used to rule out encephalitis, meningitis and encephalopathy; however, non-invasive diagnostic tests such as CT or MRI and EEG can be used in differential diagnosis for BCWG. Additionally, BCWG has a distinct disease pattern, and therefore, a careful review of the patients’ medical history and non-invasive tests are sufficient to support BCWG diagnosis [21]. In our study, we noted no abnormalities in the CT or MRI scans; and SSS in nine children. SSS are usually considered benign sporadic sleep spikes and can be observed in normal infants and toddlers, with unclear clinical significance. In summary, we do not believe BCWG clinical presentations include significant abnormalities that can be observed with CT or MRI and EEG.

## Conclusions

BCWG is often misdiagnosed as encephalitis or epilepsy, leading to unnecessary examination and treatment, which leads to increased psychological and economic burden. In this study we have reported BCWG characteristics, treatment, and favorable long-term outcomes to aid diagnosis and treatment decisions for BCWG. We recommend vaccination against rotavirus for gastroenteritis prevention, and do not recommend lumbar puncture as a diagnosis tool, or antiepileptics after discharge for the treatment of BCWG.

## Data Availability

The datasets generated and analyzed during the present study are available from the corresponding author on reasonable request.

## Abbreviations

BCWG or CwG: Benign convulsions with mild gastroenteritis
WBC: White blood cell
NE: Neutrophil elastase
LY: Lymphocyte
CRP: C-reactive protein
CK: Creatine kinase
CKMB: Creatine kinase myocardial band
HRV: Human rotavirus antigen
AG: Anion gap
Lac: Lactate
BE: Bases excess
CT: Computed tomography
MRI: Magnetic resonance imaging
CSF: Cerebrospinal fluid
EEG: Electroencephalogram
ILAE: International League Against Epilepsy
SD: Standard deviation
IQR: Interquartile range

## References

[1] Chen H, Zha J, Zhong JM, Chen Y, Yu XY, Xie JH (2019). Clinical features of campylobacter-associated benign convulsions with mild gastroenteritis compared with rotavirus convulsions. Seizure.

[2] Komori H, Wada M, Eto M, Oki H, Aida K, Fujimoto T (1995). Benign convulsions with mild gastroenteritis: a report of 10 recent cases detailing clinical varieties. Brain Dev.

[3] Morooka K (1982). Convulsions and mild diarrhea. Shonika.

[4] Kang B, Kwon YS (2014). Benign convulsion with mild gastroenteritis. Korean J Pediatr.

[5] Wu JL, Yin; Cao L, Liu R, Pang B, Dong (2002). Zuo. A study on benign infantile convulsions associated with mild gastroenteritis. Chinese Journal of Practical Pediatrics.

[6] Okumura A, Watanabe K, Negoro T (2006). Benign partial epilepsy in infancy long-term outcome and marginal syndromes. Epilepsy Res.

[7] Chen B, Cheng M, Hong S, Liao S, Ma J, Li T (2018). Clinical outcome of recurrent afebrile seizures in children with benign convulsions associated with mild gastroenteritis. Seizure.

[8] Zhang ZZ, Qihua; Lei Z, Chen R, Huang L, He G, Yuan Y, Li Z, Liu Q, Zhang C, Wang M, Liu. Xiaoyan. Re-standardization of social life ability scale for Chinese infant-junior school student. Chinese Journal of Clinical Psychology 199512–15.

[9] Lee JY, Ryu JM, Lee JS, Ko TS, Yum MS (2020). Clinical Implications of Ketosis in Children with Benign Convulsions with Mild Gastroenteritis. J Clin Neurol.

[10] Verrotti A, Nanni G, Agostinelli S, Parisi P, Capovilla G, Beccaria F (2011). Benign convulsions associated with mild gastroenteritis: a multicenter clinical study. Epilepsy Res.

[11] Ma X, Luan S, Zhao Y, Lv X, Zhang R (2019). Clinical characteristics and follow-up of benign convulsions with mild gastroenteritis among children. Med (Baltim).

[12] Khosroshahi N, Rahbarimanesh A, Boroujeni FA, Eskandarizadeh Z, Zoham MH (2018). Afebrile benign convulsion associated with mild gastroenteritis: a cohort study in a tertiary children hospital. Child Neurol Open.

[13] Verrotti A, Moavero R, Vigevano F, Cantonetti L, Guerra A, Spezia E (2014). Long-term follow-up in children with benign convulsions associated with gastroenteritis. Eur J Paediatr Neurol.

[14] Zifman E, Alehan F, Menascu S, Har-Gil M, Miller P, Saygi S (2011). Clinical characterization of gastroenteritis-related seizures in children: impact of fever and serum sodium levels. J Child Neurol.

[15] Wang YF, Zhou ZS (2013). Clinical features of benign convulsions with mild gastroenteritis in Chinese infants. World J Pediatr.

[16] You SJ. Older Patients May Have More Frequent Seizures among Children Diagnosed as Benign Convulsions with Mild Gastroenteritis. Neuropediatrics 2020. 10.1055/s-0040-1701442 32016943

[17] Li T, Hong S, Peng X, Cheng M, Jiang L (2014). Benign infantile convulsions associated with mild gastroenteritis: an electroclinical study of 34 patients. Seizure.

[18] Takami Y, Nakagawa T (2019). Efficacy of phenobarbital for benign convulsions with mild gastroenteritis: A randomized, placebo-controlled trial. Brain Dev.

[19] Kim BR, Choi GE, Kim YO, Kim MJ, Song ES, Woo YJ (2018). Incidence and characteristics of norovirus-associated benign convulsions with mild gastroenteritis, in comparison with rotavirus ones. Brain Dev.

[20] Peikes TR, Appendino JP (2014). Asymptomatic Viral Gastrointestinal Infection: The Missing Link? Pediatrics.

[21] Castellazzi L, Principi N, Agostoni C, Esposito S (2016). Benign convulsions in children with mild gastroenteritis. Eur J Paediatr Neurol.

